# Protective and Vulnerability Factors in Self-Esteem: The Role of Metacognitions, Brooding, and Resilience

**DOI:** 10.3389/fpsyg.2020.01447

**Published:** 2020-07-03

**Authors:** Roger Hagen, Audun Havnen, Odin Hjemdal, Leif Edward Ottesen Kennair, Truls Ryum, Stian Solem

**Affiliations:** Department of Psychology, Norwegian University of Science and Technology, Trondheim, Norway

**Keywords:** low self-esteem, metacognitions, depression, resilience, rumination, worry

## Abstract

The aim of the current study was to explore protective (resilience) and vulnerability factors (dysfunctional metacognitions and brooding) for self-esteem. A total of 725 participants were included in a cross-sectional study. A path analysis revealed five paths to self-esteem. The three main paths were as follows: (1) symptoms −> metacognitions −> brooding −> self-esteem, (2) symptoms −> resilience −> self-esteem, and (3) a direct path from symptoms. The first path corresponds with the metacognitive model of psychopathology and suggests that triggers in the form of anxiety and depression symptoms lead to the activation of metacognitive beliefs, which in turn activates brooding in response to these triggers. When a person engages in brooding, this makes the person vulnerable to experiencing low self-esteem. The second path suggests a protective role of resilience factors. The overall model explained 55% of the variance in self-esteem. Regression analysis found that unique predictors of self-esteem were female sex, symptoms of anxiety and depression, brooding, and resilience. These findings have possible clinical implications, as treatment may benefit from addressing both protective and vulnerability factors in individuals suffering from low self-esteem.

## Introduction

Self-esteem reflects how people feel about themselves and is a multifaceted construct related to other psychological constructs such as self-image, self-concept, self-perception, self-confidence, self-acceptance, self-respect, and self-worth. Research suggests that self-esteem is related to psychological well-being and psychological problems (Sowislo and Orth, 2013). Healthy self-esteem has been described as holding a balanced view of oneself in which one recognizes and accepts human weaknesses and appreciates ones’ strengths and good qualities (Fennel, 1998). Small, but significant, sex differences have been found, with lower levels of self-esteem among women (Kling et al., 1999).

Findings from prospective studies suggest that self-esteem is relatively stable throughout life (Orth and Robins, 2014). It is generally believed that there are many benefits to having an accepting view of the self. It seems that high self-esteem predicts success and well-being in different areas of life such as relationships, work, and health (Orth and Robins, 2014). Low self-esteem in adolescence, on the other hand, is associated with greater risk of mental health problems, substance dependence, and lower levels of life and relationship satisfaction in adulthood (Boden et al., 2008). However, the relationship between self-esteem and relevant outcomes (e.g., performance, interpersonal functioning, lifestyle, and happiness) is not always straightforward (e.g., Baumeister et al., 2003).

The association between low self-esteem and psychiatric disorders (Zeigler-Hill, 2011) indicates that low self-esteem is an important transdiagnostic construct. The association between low self-esteem and symptoms of mental disorders may be bidirectional. A meta-analysis by Sowislo and Orth (2013) found that self-esteem predicted depression, whereas the direction was unclear for anxiety disorders. Self-esteem may represent a vulnerability to problems or disorders such as depression, social anxiety, eating disorder, and substance use (Vohs et al., 1999; Donnelly et al., 2008; Sowislo and Orth, 2013), but could also be the product of psychiatric disorders. Symptoms of depression for instance may reduce self-esteem in persons with mental disorders (Shahar and Davidson, 2003; Burwell and Shirk, 2006).

The link between self-esteem and depression may be influenced by perseverative thinking processes such as rumination. Rumination, self-esteem, and depression are closely linked (Kolubinski et al., 2016). Rumination is a perseverative coping strategy in response to negative automatic thoughts. It typically consists of repeatedly contemplating past mistakes, one’s own shortcomings, negative mood, and pessimistic thoughts about the future (Wells, 2009). Self-critical rumination is defined as focusing attention specifically on self-critical thoughts and past instances of failure and seems to be linked to low self-esteem (Kolubinski et al., 2016, 2019). Moreover, cross-sectional research on the relation between self-esteem and rumination suggests that self-esteem is negatively correlated with rumination, with correlations ranging from −0.37 to −0.56 (Kuster et al., 2012). These results suggest that rumination has an important role in both development and maintenance of low self-esteem.

The self-regulatory executive functioning (S-REF) model proposed by Wells and Matthews (1994, 1996) offers a theoretical framework for how perseverative thinking could influence self-esteem. The S-REF model incorporates voluntary control of cognition, procedural knowledge (beliefs), and different levels of information processing. According to the S-REF model, emotional problems are a result of a dysfunctional response style called the cognitive attentional syndrome (CAS; Wells and Matthews, 1994). CAS is characterized by perseverative thinking such as rumination and worry, a heightened self-consciousness, threat monitoring, and other unhelpful coping strategies. Although a person’s aim with using CAS strategies is to cope with unpleasant thoughts and feelings, the strategies backfire.

The CAS is driven by metacognitive beliefs, and two important domains of metacognitive beliefs are positive and negative metacognitions. Positive metacognitions justify the activation of the CAS, such as “To analyze my flaws will help me sort out my personal problems.” Negative metacognitions concern that engaging in CAS activities is dangerous or uncontrollable, like “I can’t stop thinking negatively about myself.” From a metacognitive point of view, low self-esteem could be understood as the result of a maladaptive coping style, when a person responds to negative thoughts about oneself by ruminating about one’s flaws and shortcomings and engaging in self-criticism and negative self-talk. This response style, influenced by metacognitive beliefs, traps the individual in a vicious circle that further reinforces lower levels of self-esteem. Kolubinski et al. (2019) found that negative metacognitions, self-critical rumination, and self-criticism predicted self-esteem. Furthermore, the relationship between depression and self-esteem was partially mediated by rumination and metacognitions.

Dysfunctional metacognitions and brooding could be risk factors for low self-esteem. However, protective factors could also play an important role in self-esteem. Resilience is understood as factors that buffer against negative effects of adverse life events (Hjemdal et al., 2006). Resilience factors tap into resources that may play an important role in boosting self-esteem and protect against the development of mental health problems such as depression (Benetti and Kambouropoulos, 2006). Resilience is a multifaceted construct including the ability to have a positive outlook on life, to be goal oriented, and how one perceives oneself. Resilience is a predictor of depressive and anxiety symptoms (Hjemdal et al., 2006). One facet of resilience known as “planned future” assesses the participant’s own ability to plan future activities, goal orientation, and keeping an optimistic view of the future. Another facet, known as “perception of self,” measures confidence in one’s own abilities, self-efficacy, and having a positivistic outlook and realism in personal situations (Hjemdal, 2007).

There could be some degree of conceptual overlap (but also distinct differences) between self-esteem and the resilience factor “perception of self.” Resilience involves achieving relatively positive outcome despite experiences with adversity (Rutter, 2000), whereas self-esteem is the individual’s evaluation of one’s own value and competence. The Resilience Scale for Adults (RSA) factor “perception of self,” on the other hand, contains six items covering confidence in one’s problem-solving abilities, self-confidence, self-efficacy, having a positive outlook, realistic expectations, and accepting uncontrollable events.

It is plausible that self-esteem and perseverative thinking may affect how individuals regard their own future and plan accordingly. Research suggests that individuals reporting higher levels of resilience may have higher self-esteem than those who report lower levels and as such may be regarded as more vulnerable individuals (Dumont and Provost, 1999). To summarize, metacognitions, rumination (brooding), anxiety and depression, and resilience may be important for self-esteem. However, no studies have looked at the interplay between all these variables. The aim of the current study was therefore to investigate the relation between these variables in a large Norwegian sample.

## Materials and Methods

### Participants and Procedure

A total of 725 participants were included in the study. Participants were recruited through email lists for university students and social media. There were no set inclusion or exclusion criteria except voluntary participation. They responded to web-based questionnaires, and the survey program allowed no missing data. Participants were anonymous. Demographic information included sex (female, male), relationship status (single, partnered), and work status (student, employed). The sample was predominantly female (81.4%), 25.8% were single, and 89.9% were students or employed. Mean age was 39.6 (SD, 12.2) with ages ranging from 18 to 80 years.

### Instruments

The self-report questionnaires used are listed below.

Rosenberg Self-Esteem (RSE) Scale (Rosenberg, 1965) is a widely used measure of global self-esteem. The RSE has 10 items using a 0- to 3-point scale giving a possible range from 0 to 30. Higher scoring indicates higher degree of self-esteem. The RSE is a reliable and valid measure of global self-esteem (Gray-Little et al., 1997). Cronbach’s α was 0.89.

The Patient Health Questionnaire Anxiety–Depression Scale (PHQ-ADS; Kroenke et al., 2016) was used to assess severity of depression and anxiety symptoms. The PHQ-ADS combines the seven-item Generalized Anxiety Disorder (GAD-7; Spitzer et al., 2006) and the PHQ-9 (Kroenke et al., 2001) measures. The original GAD-7 has seven items assessing symptoms of generalized anxiety; GAD-7 uses a 0- to 3-point scale giving a possible range of 0–21. The original PHQ-9 is a nine−item self−reported questionnaire designed to evaluate the presence of depressive symptoms during the prior 2 weeks. The combined PHQ-ADS is a reliable and valid composite measure with good psychometric properties (Kroenke et al., 2016). Higher scores indicate more symptoms of anxiety and depression. Cronbach’s α was 0.93.

The Metacognitions Questionnaire (MCQ; Wells and Cartwright-Hatton, 2004) assesses metacognitions, which appear to be important factors across psychopathologies (Sun et al., 2017), and is a reliable and valid measure with good psychometric properties (e.g., Wells and Cartwright-Hatton, 2004). The MCQ has 30 items and uses a 1- to 4-point scale. Higher scores indicate more dysfunctional metacognitions. The MCQ-30 has five subscales (cognitive confidence, positive beliefs about worry, cognitive self-consciousness, negative beliefs about uncontrollability and danger, and need to control thoughts), but the total score was used in the present study. Cronbach’s α was 0.90.

The Ruminative Response Scale (RRS; Nolen-Hoeksema and Morrow, 1991) is a 22-item scale assessing the tendency to ruminate in response to depressed mood using a 1- to 4-point scale. Scores ranged from 30 to 105. The RRS has two factors: brooding and pondering/reflection (Treynor et al., 2003). Brooding involves drawing one’s attention to one’s problems and their consequences, and the current study used the five items that form the brooding subscale of the RRS. Higher scores indicate more brooding. Cronbach’s α was 0.83.

The RSA (Friborg et al., 2003; Hjemdal et al., 2006) consists of six subscales assessing resilience (perception of self, planned future, social competence, family coherence, social support, and personal structure). In the current study, we used the two subscales perception of self and planned future. These two subscales have shown the highest Cronbach’s α values and the strongest correlation with the RSA total score (Hjemdal et al., 2006). They are also the most consistent predictors of psychiatric symptoms and emotional stability (Friborg et al., 2005, 2009; Hjemdal et al., 2006). Items are scored on a 1- to 7-point scale. Higher scores indicate higher levels of resilience. Cronbach’s α was 0.91.

### Statistical Analyses

The survey program did not allow missing data. Bivariate correlations were used to explore the relationship between study variables. Then, a path analysis was conducted to investigate the role of metacognitive processes and resilience in explaining self-esteem. Hu and Bentler (1999) recommend reporting two types of fit indices: the standardized root-mean-square residual (SRMR) and a comparative fit index (CFI) such as the root-mean-square error of approximation (RMSEA). Evaluation of model fit was done according to Hu and Bentler (1999), where RMSEA < 0.06 and SRMR < 0.08 represent good fit for the model. In addition, we also reported values for the CFI and the Tucker–Lewis Index (TLI) where values close to 0.95 are needed.

Three paths were prespecified for the path analysis. The first was based on metacognitive theory in which the generic model specifies “trigger −> metacognitions −> worry/rumination −> emotional disorder” (Wells, 2009). The second path was based on theories of resilience acting as a buffer against negative life events, hence the “symptoms −> resilience −> self-esteem” path (e.g., Dumont and Provost, 1999). The third prespecified path was the direct link between symptoms and self-esteem, as previous research has suggested a close link between the two (e.g., Sowislo and Orth, 2013). These three paths were the foundation of the model upon which additional paths were included as suggested by the modification indices. Two atheoretical paths were added to improve model fit: one path from symptoms to brooding and one path from brooding to resilience. Finally, a regression analysis was used to estimate the unique predictors in explaining self-esteem.

## Results

A summary of the scores on the RSE, RSA, RRS, MCQ-30, GAD-7, PHQ-9, PHQ-ADS, and their correlations is displayed in Table 1. RSE Scale showed moderate–strong correlations with symptoms, resilience, brooding, and metacognitions. Among the MCQ-30 subscales (not included in the table), beliefs about uncontrollability and danger showed the strongest correlation with RSE (*r* = −0.46), followed by need to control thoughts (*r* = −0.39). The weakest correlation was with positive beliefs about worry (*r* = −0.21).

**TABLE 1:** Descriptive statistics and bivariate correlations among variables (*n* = 725).

	Descriptives			Correlations			
	Range	Mean	SD	2	3	4	5
1 RSE, self-esteem	15–37	28.23	4.05	0.72	–0.61	–0.47	–0.62
2 RSA, resilience	2–14	9.92	2.68		–0.70	–0.59	–0.76
3 RRS, brooding	5–20	9.50	3.24			0.64	0.69
4 MCQ-30, metacognitions	30–105	48.85	12.24				0.69
5 PHQ-ADS, symptoms	0–44	11.18	8.87				

**Note*. RSE, Rosenberg Self-Esteem Scale; RSA, Resilience Scale for Adults (subscales *perception of self* and *planned future*); RRS, Ruminative Response Scale (Subscale *Brooding*); MCQ-30, Metacognitions Questionnaire 30; PHQ-ADS, Depression and anxiety symptoms (PHQ-9 and GAD-7); GAD-7, Generalized Anxiety Disorder 7; PHQ-9, Patient Health Questionnaire 9; PHQ-ADS, Patient Health Questionnaire Anxiety–Depression Scale. All correlations were significant, *p* < 0.01.*

**FIGURE 1:**
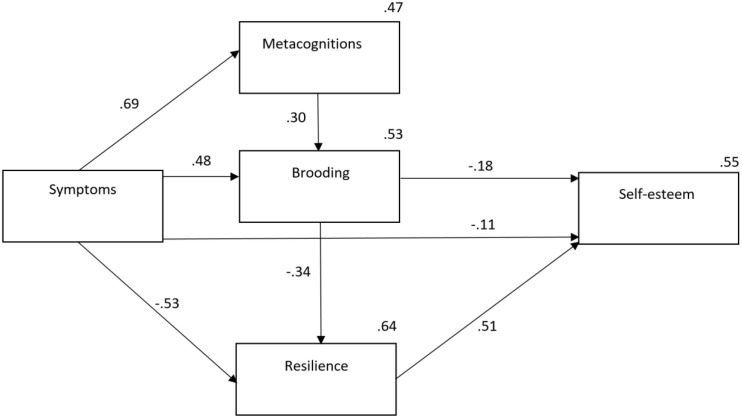
Relationship between protective and vulnerability factors for low self-esteem. *Note*. No modifications were made to the model. Symptoms, Patient Health Questionnaire Anxiety–Depression Scale.

We tested a model with metacognitions, symptoms of anxiety and depression, brooding, resilience (planned future and perception of self), and self-esteem. The fit of the proposed model was χ^2^(2) = 3.6, *p* < 0.166, SRMR = 0.0063, RMSEA.033, CFI = 0.999, TLI = 0.997, and explained 55% of the variance in self-esteem. All paths were significant (*p* < 0.001), whereas the symptoms −> self-esteem path had a *p*-value of 0.007. Five potential paths were described. The first path was as follows: symptoms −> metacognitions −> brooding −> self-esteem. The second path was as follows: symptoms −> resilience −> self-esteem. The third path was a direct path from symptoms to self-esteem. The fourth path was as follows: symptoms −> metacognitions −> brooding −> resilience −> self-esteem. The fifth and final path was as follows: symptoms −> brooding −> self-esteem. See Figure 1 for details of the model.

A regression analysis was conducted to inspect the unique variance of the study variables in explaining self-esteem. All steps added significantly to the model. Demographic variables in the first step explained 4%, whereas symptoms of anxiety and depression on Step 2 added another 35%. Metacognitions, brooding, and resilience on the following steps added another 16%. In the final model, predictors that significantly predicted self-esteem were female sex, symptoms of anxiety and depression, brooding, and resilience. See Table 2 for details.

**TABLE 2:** Summary of the hierarchical regression analyses with Rosenberg Self-Esteem Scale as the dependent variable (*n* = 725).

Step	Variable	Adj. *R*^2^	*R*^2^ cha	*F* change
1	Demographics	0.041	0.047	8.8***
2	Symptoms	0.391	0.348	413.8***
3	Metacognitions	0.393	0.003	4.2*
4	Brooding	0.455	0.062	82.6***
5	Resilience	0.550	0.094	151.9***

**Final step of the equation**		**β**	***t***	***p***

Sex		–0.079	–3.13	0.002
Age		–0.047	–1.75	0.081
Single		0.009	0.36	0.716
Working/studying		–0.008	–0.28	0.776
PHQ-ADS		–0.123	–2.77	0.006
Metacognitions		0.041	1.12	0.265
Brooding		–0.192	–4.87	0.000
Resilience		0.519	12.33	0.000

*Note*. *p < 0.05, ***p < 0.001. Symptoms, GAD-7 and PHQ-9 scores; Metacognitions, MCQ-30; PHQ-ADS, The Patient Health Questionnaire Anxiety–Depression Scale. Highest VIF value was 2.86. Lowest tolerance value was 0.35.

## Discussion

In the present study, we aimed to explore the relation between symptoms of anxiety and depression, metacognitions, brooding, resilience, and self-esteem. Metacognitions correlated with brooding, which again correlated with self-esteem. These results indicate that self-esteem is associated with perseverative thinking and underscores the importance of metacognitions and rumination for the maintenance of low self-esteem. Clinically, this makes sense as engaging in thinking about one’s flaws and shortcomings and dwelling on comparisons with other persons are likely to lead to lower self-esteem. Furthermore, participants scoring high on resilience were found to be less prone to low self-esteem. This factor comprises the ability to have a positive outlook on the future, being goal oriented, having confidence in own problem-solving abilities, and having realistic expectations. In addition, pathological brooding seemed to interact with resilience.

Theoretically, metacognitions maintain pathological brooding, which may contribute to reduced self-esteem. However, our results suggest that this association between maladaptive processes and strategies can be weakened by healthy goal-directed and future-oriented behavior and strategies that in turn can contribute to reducing negativistic mood and attitudes by leading to a more positive apprehension about future events. The results indicate that resilience is an important factor to adjust in a healthy way to negative thoughts and feelings about aspects of oneself (e.g., Dumont and Provost, 1999; Benetti and Kambouropoulos, 2006). Overall, these findings suggest that self-esteem is indeed, as suggested by Jones and Idol (1990), the result of a complex interplay between both protective and vulnerability factors.

Our study lends support to the S-REF model to explain the relation between metacognitive processes and self-esteem; metacognitive beliefs influence the degree of brooding, which again is associated with low self-esteem. The current findings dovetail nicely with recent metacognitive approaches to understanding self-esteem by Kolubinski et al. (2019).

The results of this study are potentially relevant for clinical interventions. Low self-esteem has previously been conceptualized based on the cognitive model proposed by Fennell (1997). Cognitive therapy based on schema theory and metacognitive therapy (MCT; Wells, 2009) based on the S-REF model represent different treatment approaches. Cognitive behavioral therapy (CBT) interventions are aimed at cognitive restructuring and behavioral experiments to modify schemas about one’s self and modify self-critical thoughts. CBT based on the cognitive model by Fennel (1998) has shown promising results (Kolubinski et al., 2018). However, there are few clinical trials focusing on altering low self-esteem, and only three have used a randomized controlled design (Brown et al., 2004; Waite et al., 2012; McElhinney et al., 2016).

Consequently, there are a limited number of controlled studies on psychological treatment for low-self-esteem, and existing treatments are largely based on cognitive therapy. A meta-analysis by Normann and Morina (2018) showed that for depression and anxiety disorders MCT had equal or better treatment outcome compared to CBT. Because both metacognitions and preservative thinking as shown in this study could play a prominent role in both depression and low self-esteem, MCT (that focuses directly on these constructs) could be a promising new approach.

Importantly, the results also demonstrate the importance of resilience factors. Resilience is not explicitly a part of the S-REF model (Wells and Matthews, 1994, 1996); however, these factors represent the protective, moderating effect of a person’s resources in exposure to demanding situations in life. Resilience may thus be viewed as a person’s positive coping resources. The study shows the importance of assessing a person’s coping styles, especially in terms of positive view of the future, confidence in own problem-solving abilities, having realistic expectations, and accepting uncontrollable events. This may act as a buffer for rumination and worry, while at the same time directly countering the development of low self-esteem.

A limitation of the study is its cross-sectional nature, which limits our ability to draw causal conclusions. Also, the convenience sampling procedure (and requiring all items to be answered) for recruiting participants may have influenced the response rate and representativeness of the sample. Furthermore, although the path analysis achieved good fit, this is not necessarily indicative of a good theoretical model. The model was partly data-driven as indicated with the brooding −> resilience path, in which the opposite direction could be expected. Therefore, the data suggest that brooding could influence the perception of self and future plans. Future research should therefore explore the relationship between resilience and brooding using other designs. Because of the cross-sectional design, the study did not test possible mediation effects, which should be investigated in future research using longitudinal designs. Mediation concerns hypotheses about causal processes. Mediation tests with cross-sectional data imply that causal effects are instantaneous, which is problematic (Selig and Preacher, 2009), and longitudinal and cross-sectional tests rarely yield the same results (Maxwell and Cole, 2007). Furthermore, self-esteem has been described as a sociometer designed to detect possible deleterious changes in interpersonal relations, which could prompt coping behavior aimed at protecting the person against social rejection and exclusion (e.g., Leary and Baumeister, 2000). Future research should therefore also explore alternative theoretical models including the role of associated coping behaviors and interpersonal functioning.

Another potential limitation concerns the issue of shared method variance. One limitation relates to choice of measure for metacognitions. In the current study, we used the generic MCQ, which has shown stronger relationships with worry than rumination and depression. However, results could have been somewhat different if the Metacognitions About Self-Critical Rumination Questionnaire (Kolubinski et al., 2017) had been used. Also, the tolerance value of 0.35 in the regression could suggest some multicollinearity. Future studies need to address these current limitations.

## Conclusion

The present study indicates that metacognitions, brooding, and resilience may have an effect on low self-esteem. Addressing these factors in treatment could be beneficial and should be explored in future studies.

## Data Availability Statement

The raw data supporting the conclusions of this article will be made available by the authors, without undue reservation.

## Ethics Statement

The studies involving human participants were reviewed and approved by the Regional Committee for Medical and Health Research Ethics in Norway. The patients/participants provided their written informed consent to participate in this study.

## Author Contributions

RH, OH, LK, TR, and SS were involved in study conceptualization and data collection. SS, AH, and OH analyzed the data. All authors contributed to the manuscript.

## Conflict of Interest

The authors declare that the research was conducted in the absence of any commercial or financial relationships that could be construed as a potential conflict of interest.
